# Graphene-based dual-functional chiral metamirror composed of complementary 90° rotated U-shaped resonator arrays and its equivalent circuit model

**DOI:** 10.1038/s41598-021-03457-8

**Published:** 2021-12-13

**Authors:** Somayyeh Asgari, Tapio Fabritius

**Affiliations:** Optoelectronics and Measurement Techniques Research Unit, Faculty of Information Technology and Electrical Engineering, University of Oulu, Oulu, Finland

**Keywords:** Engineering, Optics and photonics, Physics

## Abstract

An equivalent circuit model (ECM) using a MATLAB code to analyze a tunable two-layered graphene-based chiral dual-function metamirror, is proposed in this work. The investigated metastructure is composed of complementary U-shaped graphene resonator arrays in the terahertz (THz) region. The ECM analysis could be used for any two-layered chiral metastructure for any frequencies, containing resonators with a thickness less than λ/50. The characteristics of the proposed tunable metamirror were analyzed numerically using the finite element method (FEM) in CST Software to verify the ECM analysis. The proposed metamirror can be used in polarization-sensitive devices in the THz region with simpler biasing without a need for ion gels or similar. It works as a broadband TE and multiband (four bands) TM mirror in the 0.3–4.5 THz bandwidth with a strong linear dichroism (LD) response (up to 96%). The designed mirror is a dynamically tunable, dual-functional structure, requiring only 90° rotation of the incident electromagnetic fields to switch between broadband and multiband spectral behavior making it a promising candidate for future THz intelligent systems. The proposed ECM is in agreement with the FEM results. The ECM analysis provides a simple, fast, and effective way to understand the metamirror’s behavior and guides for the design and analysis of graphene-based chiral metastructures in the THz region.

## Introduction

Chiral structures do not coincide with their mirror images. Chiral metastructures are applicable platforms due to their chirality responses such as circular dichroism (CD)^1^ and/or linear dichroism (LD)^2^ in the terahertz (THz) region. Graphene-based chiral metastructures have been designed and developed recently to achieve tunable CD and/or LD chirality responses^1–6^. Graphene, 2D layer of graphite, with a thickness of 0.335 nm^7^, has become a promising material in electromagnetics and optoelectronics metastructures as it is capable of dynamically tuning electromagnetic waves^3–9^. These proposed graphene-based chiral metastructures have circular conversion dichroism (CCD) and LD up to 20% and 94%, respectively. It is highly desirable to design and introduce dynamically controllable metamirrors with dual functionality, which can show both broadband and multiband reflection spectra by altering the type of the incident wave.

Some chiral metamirrors have been introduced and designed, recently^10–15^, but none of them are dynamically controllable, dual-functional, or function in 0.3–4.5 THz frequency region, or use the proposed circuit modeling process. Dual-functional metamirrors are urgently needed in THz intelligent systems to greatly reduce the size of the THz system without needing two different metastructures to produce broadband and multiband reflections. For example, to be used in imaging objects under the broadband reflection or distinguishing them in case of multiband reflection^16^. In addition, these metasurfaces could also be favorable for other potential applications such as biosensors^17–20^, spatial modulators, attenuators^16^, and absorbers^21^.

Metastructures, containing a resonator per unit cell with single-layered geometry, have a limited number of reflection bands and broadband reflection properties. Multi-band or broadband metamirrors are typically designed by combining multiple resonators with different sizes into a super unit cell^22^ or stacking resonators as a multilayer structure with resonators with different geometrical parameters and separate with dielectric spacers^9,23^.

A few graphene-based metastructures composed of complementary patterns have been proposed to ease the biasing procedure and fabrication of graphene based metastructures^24–27^ without the need for ion gels^28^ or thin graphene strips^29^, thus reducing material usage, costs, and time in the fabrication process.

Chiral metastructures, composing of two-layered gold U-shaped rotated patterns, were previously proposed in following papers^30–32^, investigating the different aspects and applications of this configuration. However, the detailed investigation of this configuration with 2D materials like graphene is lacking. By the design, analysis and optimization of this metastructure with graphene-based complementary resonator arrays in THz region, their performance in tunable metamirror applications was investigated in this work.

In contrast to the chiral metastructure composed of common single-layered graphene-based U-shaped patterns^28^, we developed a chiral metamirror consisting of two-layered complementary U-shaped, 90° rotated, patterns. The metastructure in^28^ is a multiband absorber but the proposed structure could act as broadband and multiband metamirror just by switching the type of the incident electromagnetic waves. Such dual function surfaces are expected to be a key element of intelligent THz systems in the future since they could act as a compact broadband and a multiband mirror. Our earlier paper^28^ presented a procedure to obtain an EMC model of the metastructure but in this work we used equivalent conductivity relations and reflections to obtain the equivalent conductivities of the graphene patterns. Then, the transfer matrix elements were determined for the structure to obtain the reflection characteristics of the metasurface containing graphene/dielectric/graphene/dielectric/gold layers. In^28^, we used transfer matrix elements and reflections to obtain the impedances of the graphene layer, which were then used to achieve the transfer matrix of the whole structure containing ion gel/graphene/dielectric/gold layers to obtain the metastructure absorption behavior.

## Metastructure, material, and equivalent circuit model

**Figure 1:**
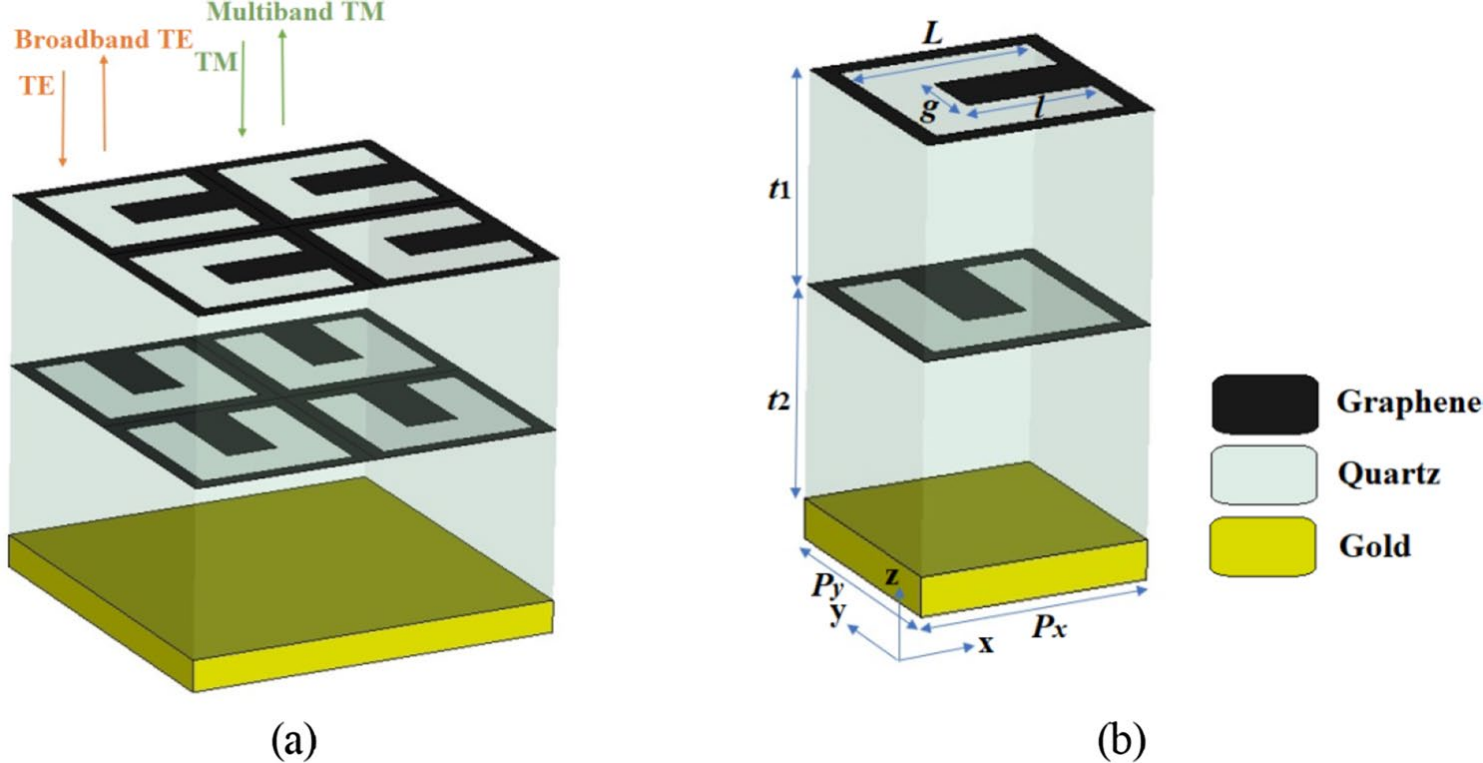
(**a**) 3D schematic view of the tunable graphene-based dual-function chiral metamirror array composed of two-layered 90° rotated complementary U-shaped resonator arrays (**b**) 3D view of the unit cell of the metamirror. The substrate and the reflector are respectively made of quartz and gold. A gold reflector is used to prevent the wave pass (the transmission is zero for both TE and TM waves).

3D schematic views of the periodic and unit cell of the proposed dynamically controllable graphene-based dual-functional chiral metamirror composed of two-layered, complementary 90° rotated U-shaped resonator arrays are respectively shown in Fig. 1a,b. In contrast to commonly used pattering^28^, the graphene patterned layers are a complementary shape in our design. Thus, there is no need to use ion gel layers for biasing the graphene layers making its tunability much easier to implement and making the fabrication procedure easier. The dielectric substrates are made of quartz with a refractive index of 1.96^33^. The metamirror metastructure is backed with a gold layer with a thickness of 0.5 μm to ensure that electromagnetic waves cannot pass through it (the transmission is zero for both TE and TM waves). The conductivity of the gold layer is 4.56 × 10^7^ S/m^34^. Simulations were performed using the CST Microwave Studio Software^4^. The device works as a broadband and multiband metamirrors when it is illuminated by TE and TM waves respectively. The structural parameters and their optimized values for the proposed dual-functional metamirror are given in Table 1. The genetic algorithm optimization technique using the CST Software was used to obtain the optimal values of the structural parameters. Genetic algorithms (GAs) are exploratory searches and optimization techniques inspired by natural evolution^35,36^. In the optimization technique in CST, we took into account that the unit cell dimensions, *P*_*x*_ = *P*_*y*_ = 18 μm, have to be smaller than λ_min_ = 66.67 μm if *f*_*max*_ = 4.5 THz (the maximum frequency in the simulated region) to avoid excitation of high order Floquet modes^37–39^.

**Table 1:** Structural parameters and their optimized values for the graphene-based chiral dual-functional metamirror of Fig. 1.

Parameter	Value	Parameter	Value	Parameter	Value
*P*_*x*_	18 μm	*P*_*y*_	18 μm	*l*	10 μm
*g*	5 μm	*L*	15 μm	*t*_1_	8 μm
*t*_2_	3 μm	–	–	–	–

For the upper graphene, *μ*_*c*_ = 1 eV and for the bottom graphene, *μ*_*c*_ = 0.95 eV are considered. The relative permittivity of graphene, by assumption of the incident electromagnetic wave as *e*^*jωt*^, is^4^:

1 $$ \varepsilon = 1 - \frac{j\sigma }{{\omega \varepsilon_{0} {\Delta }}}, $$

in which *σ*, ω, *ε*_0_, and Δ are respectively the graphene surface conductivity, angular frequency, vacuum permittivity, and graphene thickness. Δ is considered to be 0.335 nm^7^. *σ* is the sum of the inter- and intra-band electron transition contribution terms based on the Kubo formula reported in^4^.

The propagation constant for the electromagnetic wave propagation in a graphene-vacuum setup can be obtained according to^4^:

2 $$ \beta = k_{0} \sqrt {1 - \left( {\frac{2}{{\eta_{0} \sigma }}} \right)^{2} } $$

where *β*, *k*_0_, and *η*_0_ are respectively the propagation constant of the electromagnetic wave in a graphene-vacuum setup, the wave vector of the incident light wave, and the vacuum impedance.

**Figure 2:**
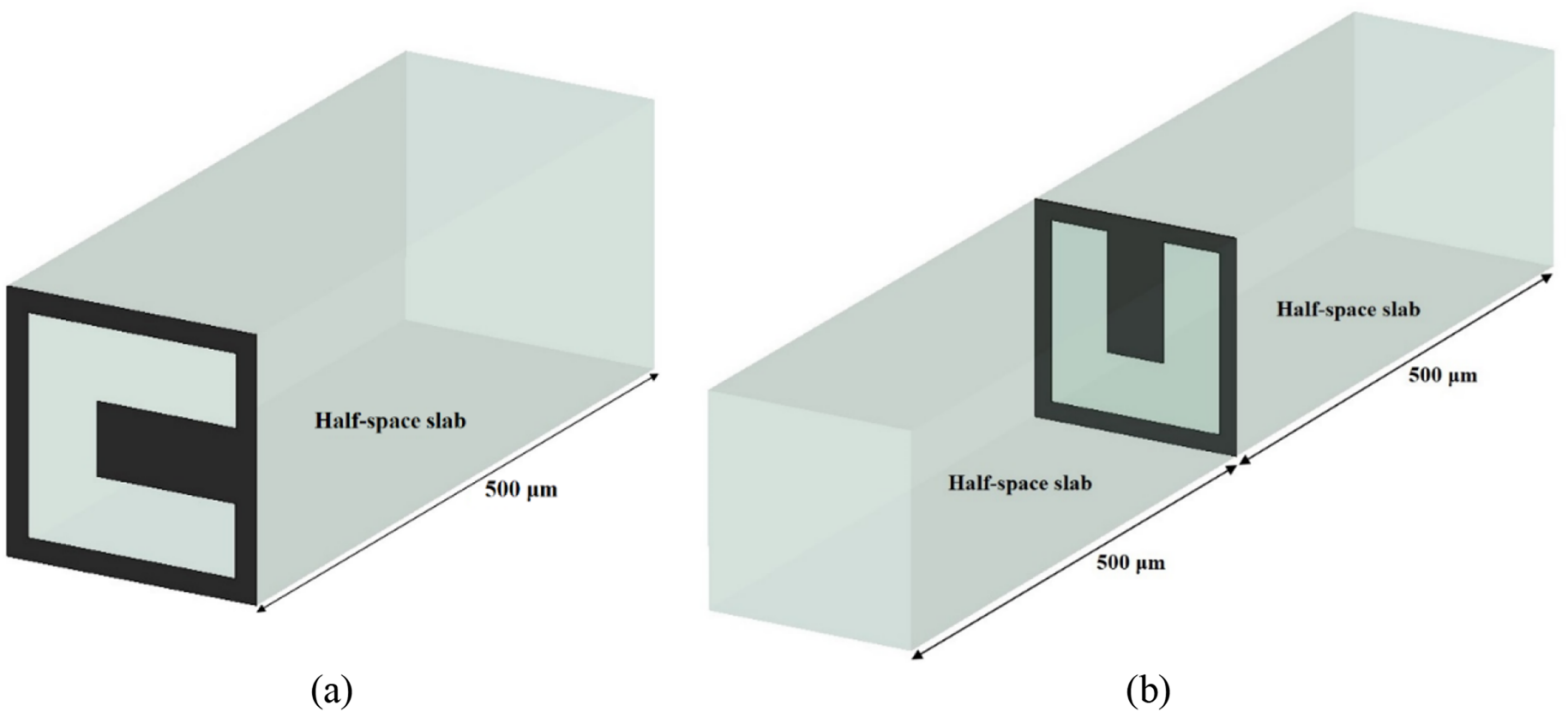
(**a**) Front and (**b**) back graphene layer setups for extraction of the equivalent conductivities of the graphene layers when they are respectively at the interfaces of air/half-space and half-space/half-space slabs.

The equivalent circuit modeling (ECM) approach to the designed dual-functional metamirror structure is based on the modeling of each patterned graphene layer as the equivalent conductivity $\sigma_{es}^{TE/TM}$. The reflection spectra *r*^*TE/TM*^ were defined by CST simulations considering the front and the back patterned graphene layers respectively on one half-space (the front patterned graphene layer in Fig. 1 is between air and dielectric) and between two half-space dielectric slabs (the back patterned graphene layer in Fig. 1 is between dielectrics, shown in Fig. 2a,b). Each half-space slab has the thickness of 500 μm.

The TE and TM reflection coefficients *r*^*TE/TM*^ for the setups considered in Fig. 2, based on Fresnel equations, are as follows^40,41^:

3 $$ r^{TE} = \frac{{\sqrt {\varepsilon_{r1} } \cos \left( {\theta_{in} } \right) - \sqrt {\varepsilon_{r2} } \cos \left( {\theta_{out} } \right) - \eta_{0} \sigma_{es}^{TE} }}{{\sqrt {\varepsilon_{r1} } \cos \left( {\theta_{in} } \right) + \sqrt {\varepsilon_{r2} } \cos \left( {\theta_{out} } \right) + \eta_{0} \sigma_{es}^{TE} }} $$

4 $$ r^{TM} = \frac{{\sqrt {\varepsilon_{r1} } \sec \left( {\theta_{in} } \right) - \sqrt {\varepsilon_{r2} } \sec \left( {\theta_{out} } \right) - \eta_{0} \sigma_{es}^{TM} }}{{\sqrt {\varepsilon_{r1} } \sec \left( {\theta_{in} } \right) + \sqrt {\varepsilon_{r2} } \sec \left( {\theta_{out} } \right) + \eta_{0} \sigma_{es}^{TM} }} $$

5 $$ \sin \left( {\theta_{out} } \right) = \sqrt {\frac{{\varepsilon_{r1} }}{{\varepsilon_{r2} }}} \sin \left( {\theta_{in} } \right) $$

in which $\varepsilon_{{r_{1} }}$, $\theta_{in}$, $\varepsilon_{{r_{2} }}$, $\theta_{out}$, and $\eta_{0}$ are respectively the relative dielectric permittivity of the front slab, the angle of the incident electromagnetic wave, the relative dielectric permittivity of the back slab, the angle of the transmitted electromagnetic wave, and the free space impedance equal to 377 Ω. By doing some algebra on Eqs. (3) and (4), $\sigma_{es}^{TE/TM}$ can be written as:

6 $$ \sigma_{es}^{TE} \left( {\omega ,\theta_{in} } \right) = \frac{{\sqrt {\varepsilon_{r1} } \cos \left( {\theta_{in} } \right) - \sqrt {\varepsilon_{r2} } \cos \left( {\theta_{out} } \right) - r^{TE} \left( {\sqrt {\varepsilon_{r1} } \cos \left( {\theta_{in} } \right) + \sqrt {\varepsilon_{r2} } \cos \left( {\theta_{out} } \right)} \right)}}{{\eta_{0} \left( {1 + r^{TE} } \right)}} $$

7 $$ \sigma_{es}^{TM} \left( {\omega ,\theta_{in} } \right) = \frac{{\sqrt {\varepsilon_{r1} } \sec \left( {\theta_{in} } \right) - \sqrt {\varepsilon_{r2} } \sec \left( {\theta_{out} } \right) - r^{TM} \left( {\sqrt {\varepsilon_{r1} } \sec \left( {\theta_{in} } \right) + \sqrt {\varepsilon_{r2} } \sec \left( {\theta_{out} } \right)} \right)}}{{\eta_{0} \left( {1 + r^{TM} } \right)}} $$

**Figure 3:**
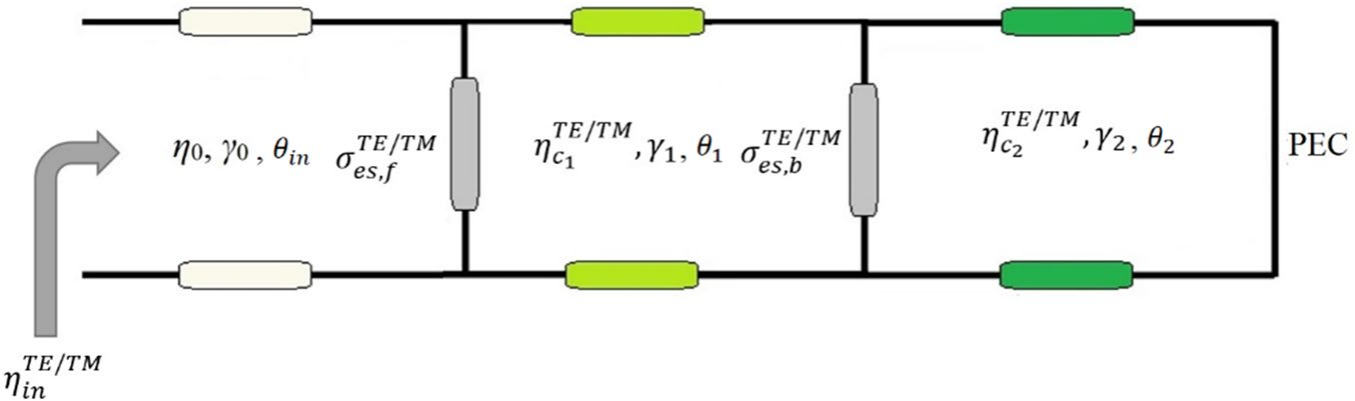
Equivalent circuit model (ECM) of the proposed graphene-based dual-function chiral metamirror.

The ECM of the introduced dual-functional chiral metamirror is presented in Fig. 3. Each patterned graphene layer is assumed to have an equivalent conductivity and each dielectric layer is considered as a transmission line. The gold layer is modeled as a perfect electric conductor (PEC) at the end of the circuit model design.

The transfer matrices of the patterned graphene layers and the dielectric layers are:

8 $$ \left[ {\phi_{{G_{i} }}^{TE/TM} } \right] = \left[ {\begin{array}{*{20}c} 1 & 0 \\ {\sigma_{{es_{i} }}^{TE/TM} } & 1 \\ \end{array} } \right] $$

9 $$ \left[ {\phi_{{d_{i} }}^{TE/TM} } \right] = \left[ {\begin{array}{*{20}c} {\cosh \left( {\gamma_{i} t_{i} } \right)} & {\eta_{{c_{i} }}^{TE/TM} \sinh \left( {\gamma_{i} t_{i} } \right)} \\ {\frac{1}{{\eta_{{c_{i} }}^{TE/TM} }}\sinh \left( {\gamma_{i} t_{i} } \right)} & {\cosh \left( {\gamma_{i} t_{i} } \right)} \\ \end{array} } \right] $$

in which $\gamma_{i}$, $t_{i}$, and $\eta_{{c_{i} }}^{TE/TM}$ are respectively the wavenumber, the thickness of dielectric layer, and the dielectric layer impedance. The total transfer matrix of the proposed metamirror is:

10 $$ \left[ {\phi_{tot}^{TE/TM} } \right] = \left[ {\phi_{{G_{1} }}^{TE/TM} } \right] \times \left[ {\phi_{{d_{1} }}^{TE/TM} } \right] \times \left[ {\phi_{{G_{2} }}^{TE/TM} } \right] \times \left[ {\phi_{{d_{2} }}^{TE/TM} } \right] $$

which is equal to:

11 $$ \left[ {\phi_{tot}^{TE/TM} } \right] = \left[ {\begin{array}{*{20}c} {\phi_{tot}^{TE/TM} \left( {1,1} \right)} & {\phi_{tot}^{TE/TM} \left( {1,2} \right)} \\ {\phi_{tot}^{TE/TM} \left( {2,1} \right)} & {\phi_{tot}^{TE/TM} \left( {2,2} \right)} \\ \end{array} } \right] $$

The matrix components are calculated and given as follows:

12 $$ \begin{aligned} \phi_{tot}^{TE/TM} \left( {1,1} \right) & = \cosh \left( {\gamma_{2} t_{2} } \right)\left[ {\cosh \left( {\gamma_{1} t_{1} } \right) + \eta_{{c_{1} }}^{TE/TM} \sigma_{{es_{2} }}^{TE/TM} \sinh \left( {\gamma_{1} t_{1} } \right)} \right] \\ & \quad + \left[ {\frac{{\eta_{{c_{1} }}^{TE/TM} }}{{\eta_{{c_{2} }}^{TE/TM} }}\sinh \left( {\gamma_{1} t_{1} } \right)\sinh \left( {\gamma_{2} t_{2} } \right)} \right] \\ \end{aligned} $$

13 $$ \begin{aligned} \phi_{tot}^{TE/TM} \left( {1,2} \right) & = \eta_{{c_{2} }}^{TE/TM} \sinh \left( {\gamma_{2} t_{2} } \right)\left[ {\cosh \left( {\gamma_{1} t_{1} } \right) + \eta_{{c_{1} }}^{TE/TM} \sigma_{{es_{2} }}^{TE/TM} \sinh \left( {\gamma_{1} t_{1} } \right)} \right] \\ & \quad + \left[ {\eta_{{c_{1} }}^{TE/TM} \sinh \left( {\gamma_{1} t_{1} } \right)\cosh \left( {\gamma_{2} t_{2} } \right)} \right] \\ \end{aligned} $$

14 $$ \begin{aligned} \phi_{tot}^{TE/TM} \left( {2,1} \right) & = \cosh \left( {\gamma_{2} t_{2} } \right)\left[ \begin{gathered} \sigma_{{es_{1} }}^{TE/TM} \cosh \left( {\gamma_{1} t_{1} } \right) + \frac{1}{{\eta_{{c_{1} }}^{TE/TM} }}\sinh \left( {\gamma_{1} t_{1} } \right) + \hfill \\ \sigma_{{es_{1} }}^{TE/TM} \sigma_{{es_{2} }}^{TE/TM} \eta_{{c_{1} }}^{TE/TM} \sinh \left( {\gamma_{1} t_{1} } \right) \hfill \\ + \sigma_{{es_{2} }}^{TE/TM} \cosh \left( {\gamma_{1} t_{1} } \right) \hfill \\ \end{gathered} \right] \\ & \quad + \frac{1}{{\eta_{{c_{2} }}^{TE/TM} }}\sinh \left( {\gamma_{2} t_{2} } \right)\left[ {\sigma_{{es_{1} }}^{TE/TM} \eta_{{c_{1} }}^{TE/TM} \sinh \left( {\gamma_{1} t_{1} } \right) + \cosh \left( {\gamma_{1} t_{1} } \right)} \right] \\ \end{aligned} $$

15 $$ \begin{aligned} \phi_{tot}^{TE/TM} \left( {2,2} \right) & = \eta_{{c_{2} }}^{TE/TM} \sinh \left( {\gamma_{2} t_{2} } \right)\left[ \begin{gathered} \sigma_{{es_{1} }}^{TE/TM} \cosh \left( {\gamma_{1} t_{1} } \right) + \frac{1}{{\eta_{{c_{1} }}^{TE/TM} }}\sinh \left( {\gamma_{1} t_{1} } \right) + \hfill \\ \sigma_{{es_{1} }}^{TE/TM} \sigma_{{es_{2} }}^{TE/TM} \eta_{{c_{1} }}^{TE/TM} \sinh \left( {\gamma_{1} t_{1} } \right) + \hfill \\ \sigma_{{es_{2} }}^{TE/TM} \cosh \left( {\gamma_{1} t_{1} } \right) \hfill \\ \end{gathered} \right] \\ & \quad + \cosh \left( {\gamma_{2} t_{2} } \right)\left[ {\sigma_{{es_{1} }}^{TE/TM} \eta_{{c_{1} }}^{TE/TM} \sinh \left( {\gamma_{1} t_{1} } \right) + \cosh \left( {\gamma_{1} t_{1} } \right)} \right] \\ \end{aligned} $$

The input impedance of the chiral metamirror is calculated by:

16 $$ \eta_{in}^{TE/TM} = \frac{{\phi_{tot}^{TE/TM} \left( {1,2} \right)}}{{\phi_{tot}^{TE/TM} \left( {2,2} \right)}} $$

The scattering parameter $S_{11}^{TE/TM}$ is calculated by:

17 $$ S_{11}^{TE} = \frac{{\eta_{in}^{TE} - \eta_{0} \cos \left( {\theta_{inc} } \right)}}{{\eta_{in}^{TE} + \eta_{0} \cos \left( {\theta_{inc} } \right)}} $$

18 $$ S_{11}^{TM} = \frac{{\eta_{in}^{TM} - \eta_{0} \sec \left( {\theta_{inc} } \right)}}{{\eta_{in}^{TM} + \eta_{0} \sec \left( {\theta_{inc} } \right)}} $$

Additionally, $\eta_{{c_{1} }}^{TE}$, $\eta_{{c_{2} }}^{TE}$, $\eta_{{c_{1} }}^{TM}$, $\eta_{{c_{2} }}^{TM}$, $\gamma_{1}$, and $\gamma_{2}$ are calculated by:

19 $$ \eta_{{c_{1} }}^{TE} = \frac{{\eta_{0} }}{{\sqrt {\varepsilon_{{r_{1} }} } }}\cos \left( {\theta_{1} } \right) $$

in which

20 $$ \theta_{1} = \sin^{ - 1} \left( {\frac{{\sin \left( {\theta_{in} } \right)}}{{\sqrt {\varepsilon_{{r_{1} }} } }}} \right) $$

So,

21 $$ \eta_{{c_{1} }}^{TE} = \frac{{\eta_{0} }}{{\sqrt {\varepsilon_{{r_{1} }} } }}\cos \left( {\sin^{ - 1} \left( {\frac{{\sin \left( {\theta_{in} } \right)}}{{\sqrt {\varepsilon_{{r_{1} }} } }}} \right)} \right) $$

22 $$ \eta_{{c_{2} }}^{TE} = \frac{{\eta_{0} }}{{\sqrt {\varepsilon_{r2} } }}\cos \left( {\theta_{2} } \right) $$

in which

23 $$ \theta_{2} = \sin^{ - 1} \left( {\frac{{\sin \left( {\theta_{in} } \right)}}{{\sqrt {\varepsilon_{{r_{2} }} } }}} \right) $$

So,

24 $$ \eta_{{c_{2} }}^{TE} = \frac{{\eta_{0} }}{{\sqrt {\varepsilon_{{r_{2} }} } }}\cos \left( {\sin^{ - 1} \left( {\frac{{\sin \left( {\theta_{in} } \right)}}{{\sqrt {\varepsilon_{{r_{2} }} } }}} \right)} \right) $$

25 $$ \eta_{{c_{1} }}^{TM} = \frac{{\eta_{0} }}{{\sqrt {\varepsilon_{{r_{1} }} } }}\sec \left( {\theta_{1} } \right) $$

So,

26 $$ \eta_{{c_{1} }}^{TM} = \frac{{\eta_{0} }}{{\sqrt {\varepsilon_{{r_{1} }} } }}\sec \left( {\sin^{ - 1} \left( {\frac{{\sin \left( {\theta_{in} } \right)}}{{\sqrt {\varepsilon_{{r_{1} }} } }}} \right)} \right) $$

27 $$ \eta_{{c_{2} }}^{TM} = \frac{{\eta_{0} }}{{\sqrt {\varepsilon_{{r_{2} }} } }}\sec \left( {\theta_{2} } \right) $$

So,

28 $$ \eta_{{c_{2} }}^{TM} = \frac{{\eta_{0} }}{{\sqrt {\varepsilon_{{r_{2} }} } }}\sec \left( {\sin^{ - 1} \left( {\frac{{\sin \left( {\theta_{in} } \right)}}{{\sqrt {\varepsilon_{{r_{2} }} } }}} \right)} \right) $$

29 $$ \gamma_{1} = j\omega \sqrt {\varepsilon_{1} \mu_{1} } \cos \left( {\theta_{1} } \right) $$

So,

30 $$ \gamma_{1} = j\omega \sqrt {\varepsilon_{1} \mu_{1} } \cos \left( {\sin^{ - 1} \left( {\frac{{\sin \left( {\theta_{in} } \right)}}{{\sqrt {\varepsilon_{{r_{1} }} } }}} \right)} \right) $$

31 $$ \gamma_{2} = j\omega \sqrt {\varepsilon_{2} \mu_{2} } \cos \left( {\theta_{2} } \right) $$

So,

32 $$ \gamma_{2} = j\omega \sqrt {\varepsilon_{2} \mu_{2} } \cos \left( {\sin^{ - 1} \left( {\frac{{\sin \left( {\theta_{in} } \right)}}{{\sqrt {\varepsilon_{{r_{2} }} } }}} \right)} \right) $$

Finally, the reflection coefficients *R*^*TE/TM*^ of the dual-functional chiral metamirror is calculated by:

33 $$ R^{TE/TM} = \left| {S_{11}^{TE/TM} } \right|^{2} $$

## Results and discussion

Numerical simulations were done using the finite element method (FEM) in the frequency domain solver of CST 2018^4,42^. The boundary conditions and the mesh type are considered the same as in Ref.^4^. The metamirror was excited by TE and TM electromagnetic waves in the z-direction.

**Figure 4:**
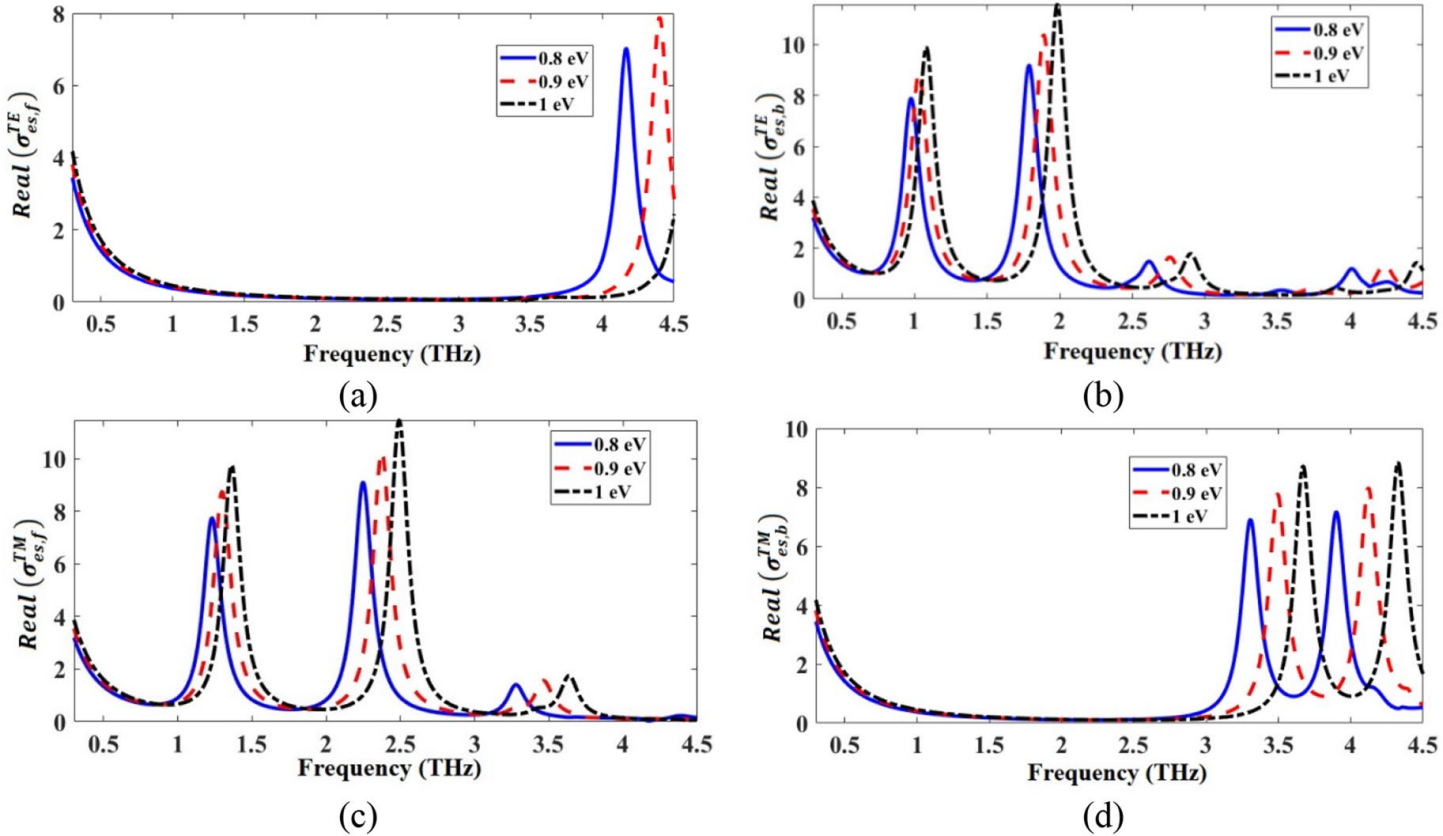
Real parts of (**a**) ${\sigma }_{es, f}^{TE}$ (conductivity of the front graphene layer in TE mode), (**b**) ${\sigma }_{es, b}^{TE}$ (conductivity of the back graphene layer in TE mode), (**c**) ${\sigma }_{es, f}^{TM}$(conductivity of the front graphene layer in TM mode), and (**d**) ${\sigma }_{es, b}^{TM}$ (conductivity of the back graphene layer in TM mode).

**Figure 5:**
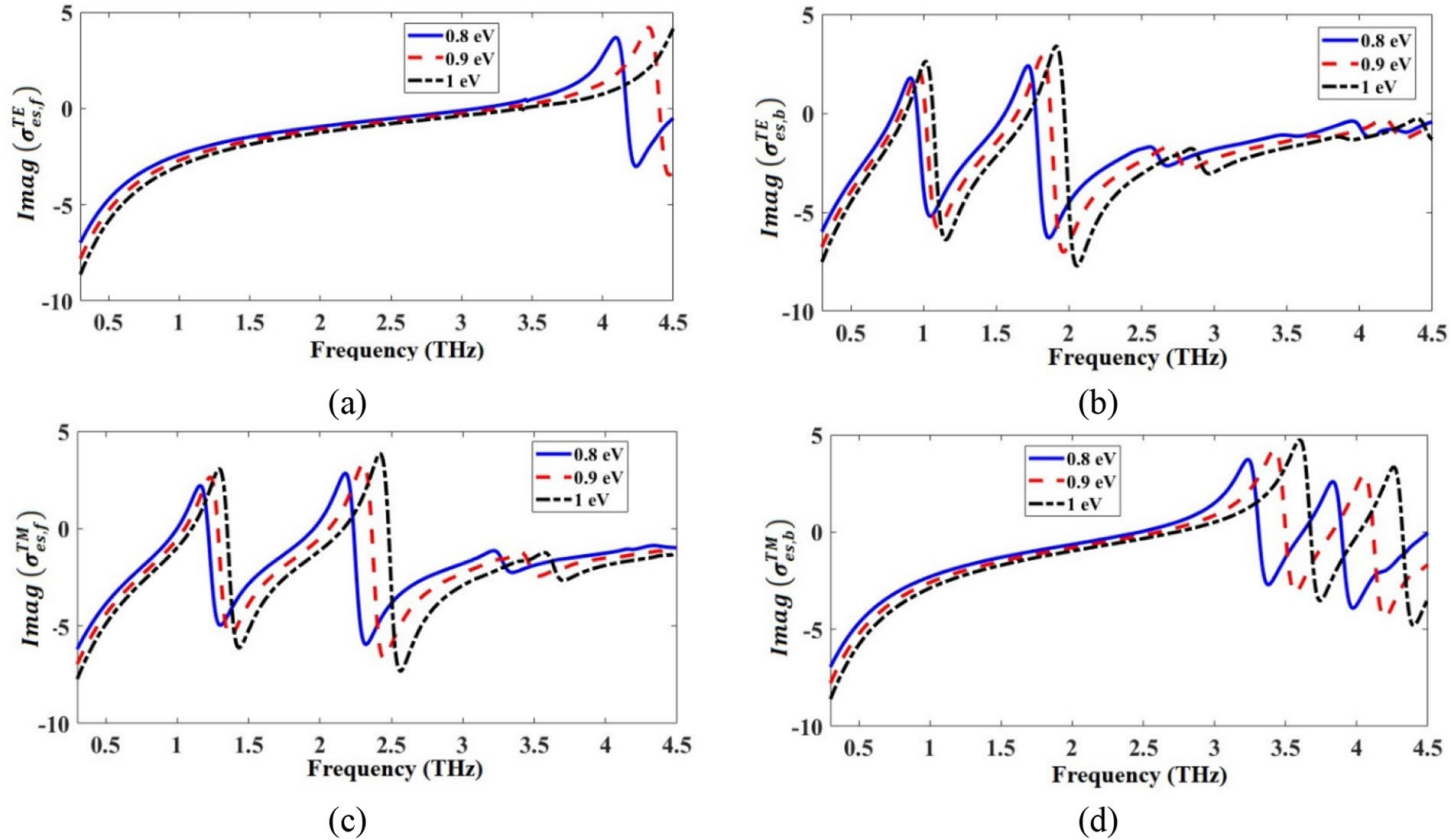
Imaginary parts of (**a**) ${\sigma }_{es, f}^{TE}$ (conductivity of the front graphene layer in the TE mode), (**b**) ${\sigma }_{es, b}^{TE}$ (conductivity of the back graphene layer in the TE mode), (**c**) ${\sigma }_{es, f}^{TM}$ (conductivity of the front graphene layer in TM mode), and (**d**) ${\sigma }_{es, b}^{TM}$ (conductivity of the back graphene layer in TM mode).

The TE/TM equivalent conductivities of the front and the back patterned graphene layers $\sigma_{es,f}^{TE/TM}$ and $\sigma_{es,b}^{TE/TM}$ were calculated by simulating the structures in Fig. 2a,b and by using Eqs. (6) and (7). The real and the imaginary parts of the calculated equivalent conductivities of the patterned graphene layers are respectively given in Figs. 4 and 5. As shown in Fig. 4, the positive real parts of the equivalent conductivities indicate the origin of loss, representing the resistive nature of the patterned graphene layers. Additionally, as it is shown in Fig. 5, the imaginary parts of the equivalent conductivities contain both positive and negative parts, respectively indicating the inductive and capacitive nature of the patterned graphene layers. As a result, each graphene layer could be modeled as a series RLC circuit. By increasing the *μ*_*c*_, the resonance frequencies of the real and the imaginary parts of $\sigma_{es,f}^{TE/TM}$ and $\sigma_{es,b}^{TE/TM}$ of the absorber increase, which tends to exhibit a blueshift. This is because the real part of the *β* in Eq. (2) decreases as the *μ*_*c*_ increases^4,43^, so, the resonance values of the real and the imaginary parts of the impedances increase by the increase of *μ*_*c*_.

**Figure 6:**
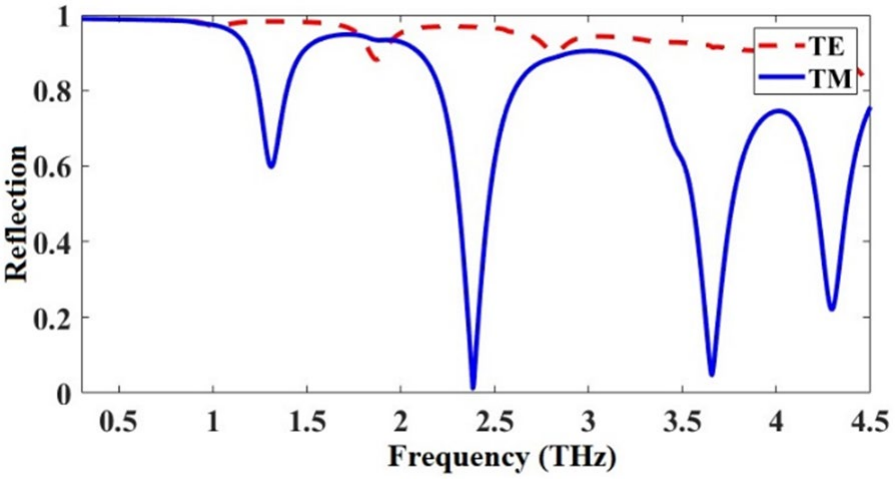
Reflection spectra of the proposed graphene-based dual-function chiral metamirror of Fig. 1 considering *μ*_*c*_ = 1 eV for the upper graphene and 0.95 eV for the bottom graphene, for TE and TM modes.

**Figure 7:**
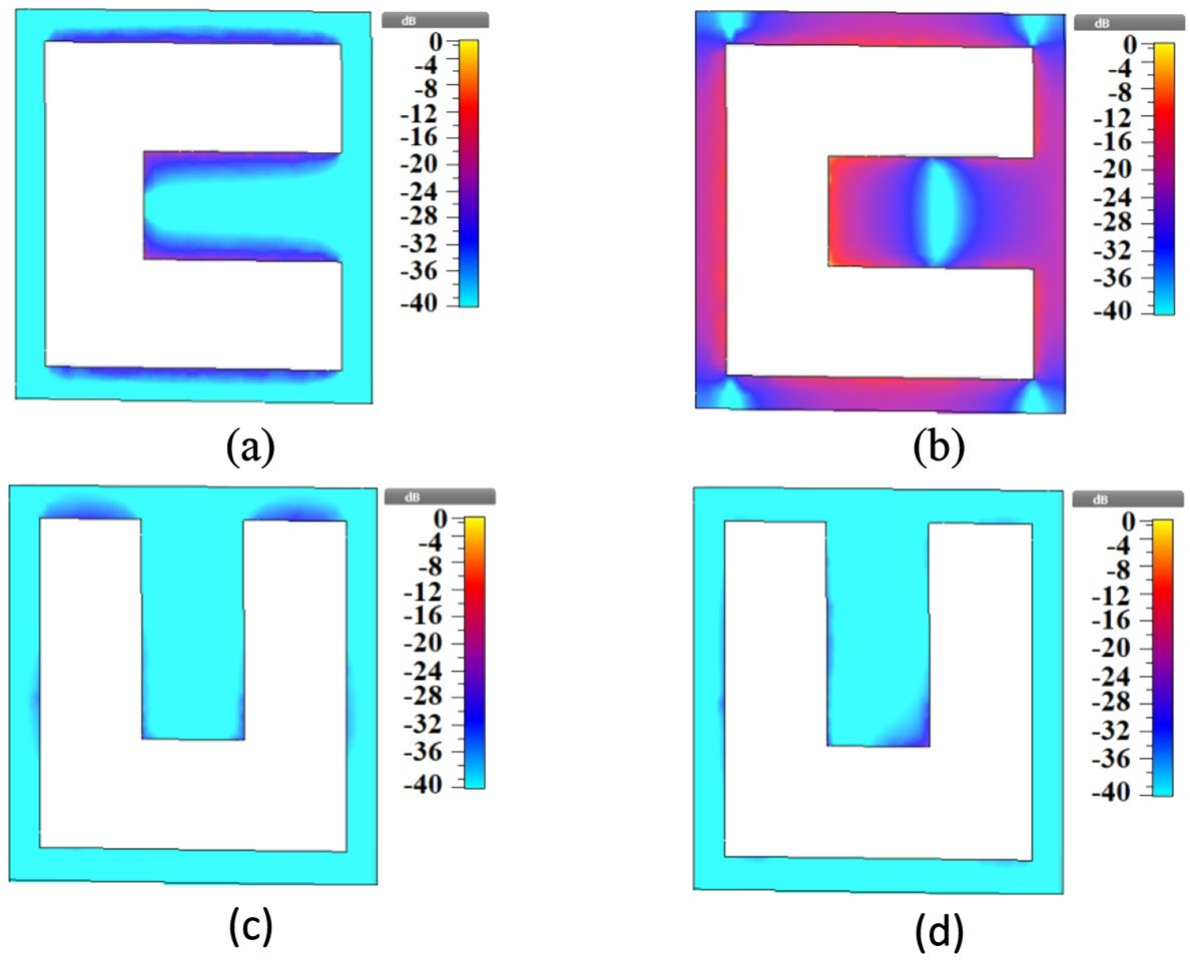
Electric field distributions of the proposed graphene-based chiral metamirror of Fig. 1 for (**a**) TE (front layer), (**b**) TM (front layer), (**c**) TE (back layer), and (**d**) TM (back layer) in 2.38 THz.

The TE/TM reflection spectra of the proposed metamirror metastructure of Fig. 1 were obtained in CST and presented in Fig. 6. As an interesting observation, the structure is dual-functional: it has a broadband reflection for the TE mode and a multiband reflection for the TM mode in the 0.3–4.5 THz frequencies. The electric field distributions of the metamirror were obtained in one of the resonances, 2.38 THz, to show the chirality nature of the device also via electric field distributions and the results are given in Fig. 7. For example, as shown in Fig. 7a,b, the front layer does not have equal distributions for TE and TM wave illuminations, respectively. It is clear that the chiral nature (asymmetric geometry and lack of mirror symmetry) of the metamirror of Fig. 1 causes these differences (the same as the electric field distributions in Fig. 7c,d).

**Figure 8:**
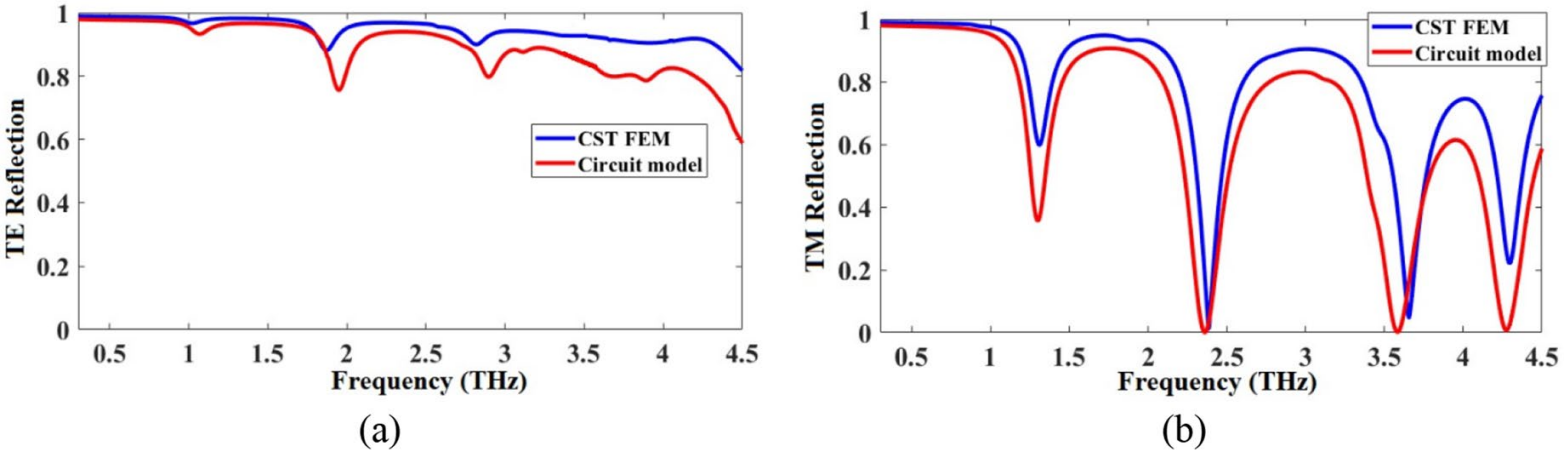
Comparison of FEM and ECM results of the proposed graphene-based chiral metamirror for (**a**) TE and (**b**) TM modes.

The TE/TM reflection spectra of the metamirror metastructure of Fig. 1 obtained by numerical simulation in CST and Eq. (33) by the theoretical ECM approach in MATLAB are given and compared in Fig. 8. As shown in those figures, the obtained results by those two different methods are in good agreement.

**Figure 9:**
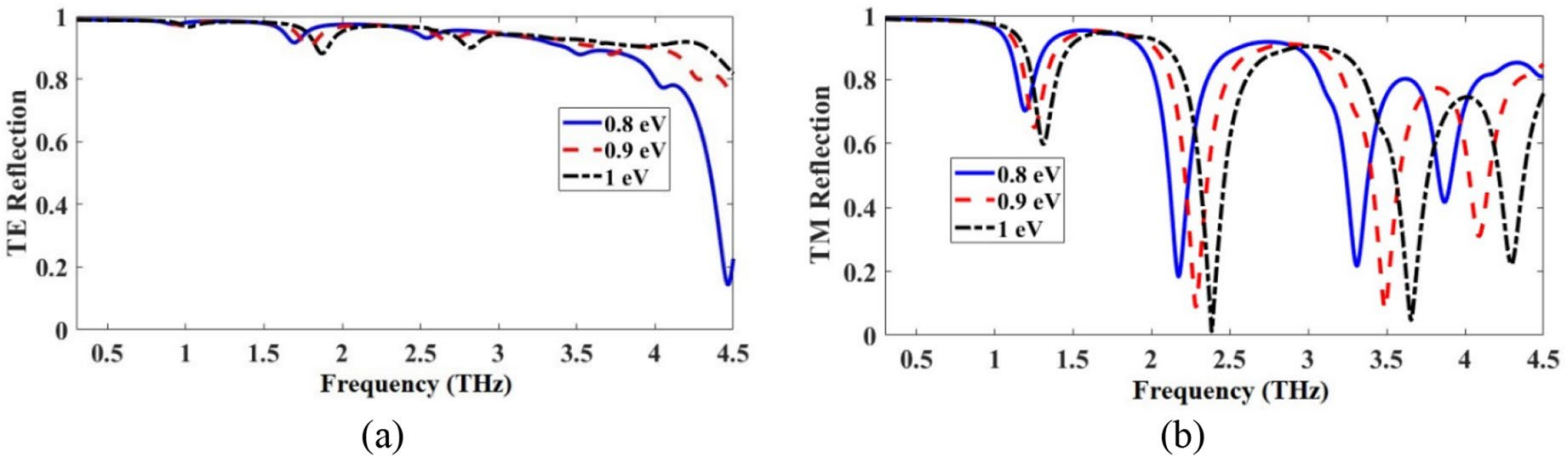
Reflection spectra of the metamirror from Fig. 1 for different values of *μ*_*c*_ for (**a**) TE and (**b**) TM modes.

The TE/TM reflection spectra of the proposed metamirror of Fig. 1 for three different values of *μ*_*c*_ are given in Fig. 9. The reflection spectra of the metamirror could be dynamically controlled by the alternation of the applied bias voltage to graphene layers. This feature is highly beneficial in graphene-based devices saving costs, materials and time as there is no need to refabricate the structure for furture experiments. By increasing *μ*_*c*_, the resonance frequencies of the absorber increase, which tends to exhibit a blueshift. This is because the real part of the *β* in Eq. (2) decreases as the *μ*_*c*_ increases^4,43^. So, the resonance values of the absorber increase by the increase of *μ*_*c*_. The structure acts as a broadband and multiband metamirror respectively for TE and TM incident electromagnetic waves in the 0.3–4.5 THz frequency region.

**Figure 10:**
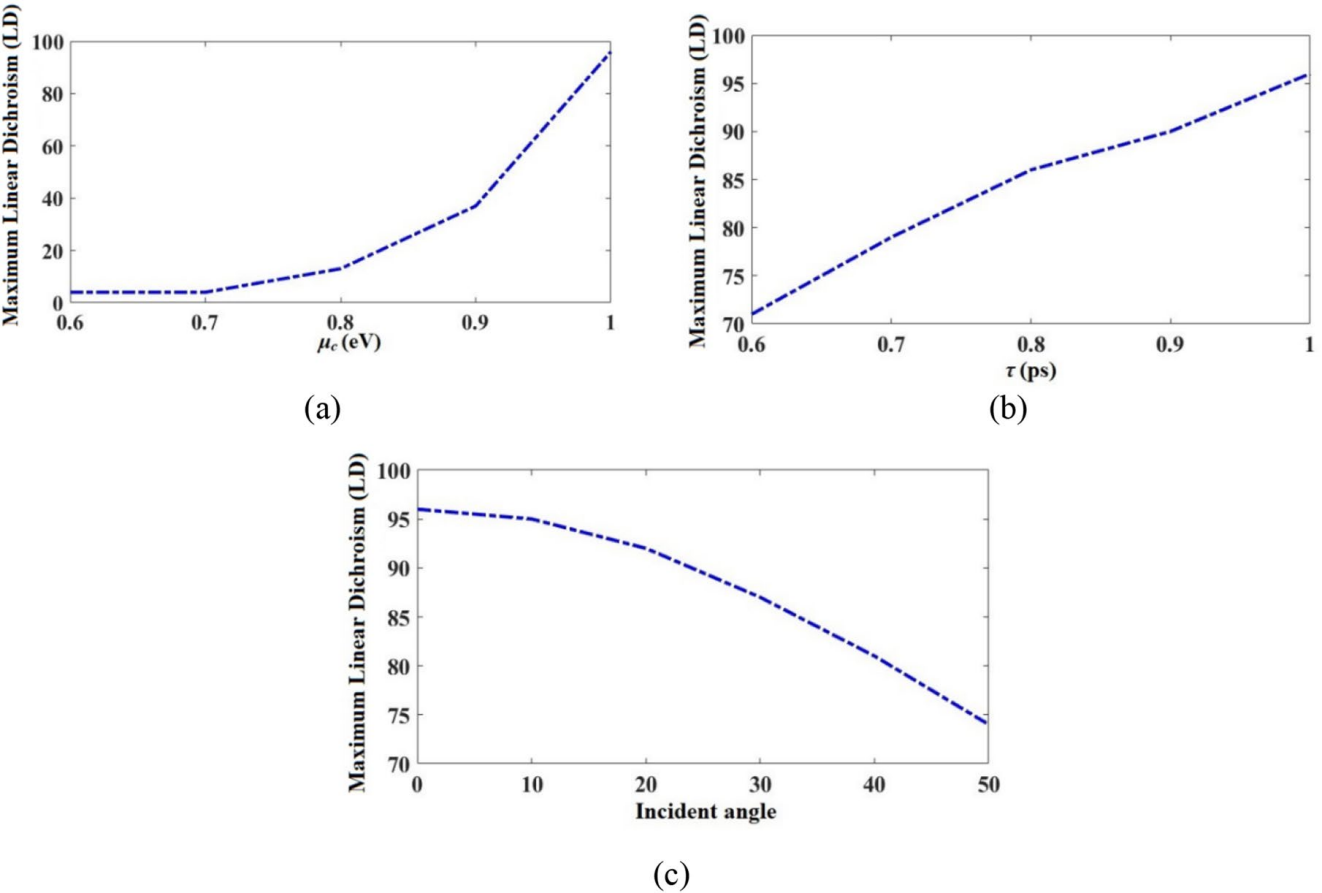
(**a**) Maximum LD vs *μ*_*c*_ spectrum of the metamirror, considering *τ* = 1 ps. The horizontal axis is the *μ*_*c*_ of the upper graphene and the *μ*_*c*_ of the bottom graphene in each step is 0.05 lower in value than it is for the upper graphene. (**b**) Maximum LD vs *τ* spectrum of the metamirror, considering *μ*_*c*_ = 1 eV for the upper graphene and 0.95 eV for the bottom graphene. (**c**) Maximum LD vs incident angle spectrum of the metamirror *μ*_*c*_ = 1 eV for the upper graphene and 0.95 eV for the lower graphene, and *τ* = 1 ps.

The maximum linear dichroism (LD difference between TE and TM absorption/reflection spectra^44^) vs *μ*_*c*_ spectrum for the metamirror is given in Fig. 10a. As shown, it is possible to dynamically tune the LD values by changing the applied bias voltage to the graphene. Chiral biomolecules such as DNA and amino acids have a weak chirality response^45^. Chiral metastructures exhibit strong interaction with light and they can improve the chirality response of biomolecules by orders of magnitude^46^. Using of graphene-based chiral metastructures, it is possible to dynamically tune the chirality response which can greatly improve the detection sensitivity^3^. By the increasing *μ*_*c*_, the maximum LD value increases. The maximum obtained LD is 96% occurred for *μ*_*c*_ = 1 eV for the upper graphene and 0.95 eV for the bottom graphene. Additionally, the maximum LD vs *τ* and vs *θ*_*in*_ are respectively given in Fig. 10a,b. It is clear that by changing the *τ* or *θ*_*in*_, the maximum LD could be tuned without a need to refabricate the metamirror. By increasing *τ*, the maximum LD value increases. By increasing *θ*_*in*_, the maximum LD value decreases. As a conclusion from Fig. 10a–c, the maximum LD for the metamirror structure is obtained with *μ*_*c*_ = 1 eV for upper graphene and 0.95 eV for the bottom graphene, *τ* = 1 ps, and *θ*_*in*_ = 0°.

The proposed chiral metamirror was compared with previously published chiral metamirrors in Table 2. Our metamirror has two valuable and advantageous features. It is dynamically tunable and dual-functional. It works as a broadband and a multiband mirror respectively for TE and TM incident electromagnetic waves in the 0.3–4.5 THz frequency region. In contrast to previously designed multiband/broadband graphene absorbers^16,47–49^, our proposed chiral metadevice can switch from multiband reflection to broadband reflection with a 90° rotation of the incident electromagnetic fields, which is an advantage compared to the others^16,47–49^. Because in Refs.^16,47–49^, in order to switch from multiband spectrum to broadband spectrum, the proposed absorbers used phase change materials (such as VO_2_)^16^ or they have to be refabricated with different dimensions^47–49^. This has potential applications in THz intelligent systems by greatly reducing the system size and could act as a broadband and multiband metamirror by just alternating the incident electromagnetic wave from TE to TM. Our proposed metastructure could be used for imaging objects under the broad-band reflection or distinguishing them in case of multi-band reflection^16^.

**Table 2:** Comparison of our proposed metamirror with previously published metamirrors.

	Dynamic tunability	Dual-functional	Frequency region	Circuit model	Max reflection (%)	Max. chirality response (%)
^10^	No	No	9.1–11 GHz	No	98	CD/93
^11^	No	No	300–375 THz	No	90	CCD/43
^12^	No	No	30–50 THz	No	99	CD/94
^13^	No	No	285–425 THz	No	90	CD/50
^14^	No	No	211–227 THz	No	80	CD/63
^15^	No	No	8–12 GHz	No	95	CD/88
This work	Yes	Yes	0.3–4.5 THz	Yes	99	LD/96

The fabrication of a structure containing two graphene layers is more difficult than the fabrication of a device containing a single graphene layer. However, the fabrication of multilayer graphene structures has been shown to be possible^50,51^ at least for the complementary pattern design, which does not require ion gel to properly bias the graphene pattern. The fabrication steps of the proposed chiral metamirror could be done as follows: (1) the bottom quartz dielectric is deposited on a gold metal sheet through thermal evaporation, (2) the bottom graphene layer is coated on the quartz by chemical vapor deposition (CVD), (3) the bottom graphene pattern is written by electron beam etching, (4) the upper quartz layer is transferred on the combination by thermal evaporation, (5) the upper graphene layer is coated on the upper quartz by CVD, and (6) the upper graphene pattern is written by electron beam etching^52,53^.

The proposed metamaterial model assumes that all the layers are fabricated without any defects. The fabrication of the proposed device and its influence on metamaterial performance needs further investigation.

## Conclusion

In this paper, an equivalent circuit modeling (ECM) approach to a tunable graphene-based dual-functional chiral metamirror composed of complementary 90° rotated U-shaped resonator arrays by using simple and fast MATLAB code was proposed, designed, and analyzed in the terahertz (THz) region. The simulation results were done using the finite element method (FEM) in CST Microwave Studio Software and were in good agreement with the ECM ones. The proposed ECM approach could be used for modeling other two-layered chiral metastructures for any frequencies, containing resonators with a thickness in order of less than λ/50. Our metamirror device is tunable, and it has strong linear dichroism (LD) response of 96%. It is a dynamically tunable, dual-functional mirror in the 0.3–4.5 THz range which makes it very promising for future THz intelligent systems. Using one device to achieve two different functions can greatly reduce the size of terahertz system thus saving material, costs, and time as there is no need to use a phase change material such as VO_2_ or to refabricate the structure with new dimensions for switching between broadband and multiband reflection spectra. The reported structure acts as a broad-band TE mirror and multi-band TM mirror but with a 90° rotation of the structure, it could act as a broad-band TM mirror and a multi-band TE mirror. Additionally, the proposed metastructure could find some other potential applications in tunable polarization sensitive structures in the future.
